# Predicting candidate therapeutic drugs for sepsis-induced acute respiratory distress syndrome based on transcriptome profiling

**DOI:** 10.1080/21655979.2021.1917981

**Published:** 2021-04-27

**Authors:** Jiawei Ma, Qianqian Li, Dandan Ji, Liang Luo, Lei Hong

**Affiliations:** a Department of Critical Care Medicine, The Affiliated Wuxi No.2 People’s Hospital of Nanjing Medical University, Wuxi, China; b Institute of Clinical Medicine Research, The Affiliated Suzhou Science and Technology Town Hospital of Nanjing Medical University, Suzhou, China

**Keywords:** Sepsis-induced ARDS, functional enrichment analysis, protein-protein interaction (PPI), connectivity map, molecular docking

## Abstract

Sepsis-induced acute respiratory distress syndrome (ARDS) remains a major threat to human health without effective therapeutic drugs. Previous studies demonstrated the power of gene expression profiling to reveal pathological changes associated with sepsis-induced ARDS. However, there is still a lack of systematic data mining framework for identifying potential targets for treatment. In this study, we demonstrated the feasibility of druggable targets prediction based on gene expression data. Through the functional enrichment analysis of microarray-based expression profiles between sepsis-induced ARDS and non-sepsis ARDS samples, we revealed genes involved in anti-microbial infection immunity were significantly altered in sepsis-induced ARDS. Protein–protein interaction (PPI) network analysis highlighted TOP2A gene as the key regulator in the dysregulated gene network of sepsis-induced ARDS. We were also able to predict several therapeutic drug candidates for sepsis-induced ARDS using Connectivity Map (Cmap) database, among which doxorubicin was identified to interact with TOP2A with a high affinity similar to its endogenous ligand. Overall, our findings suggest that doxorubicin could be a potential therapeutic for sepsis-induced ARDS by targeting TOP2A, which requires further investigation and validation. The whole study relies on publicly available dataset and publicly accessible database or bioinformatic tools for data mining. Therefore, our study benchmarks a workflow for druggable target prediction which can be widely applicable in the search of targets in other pathological conditions.

## Graphical Abstract

**Figure:**
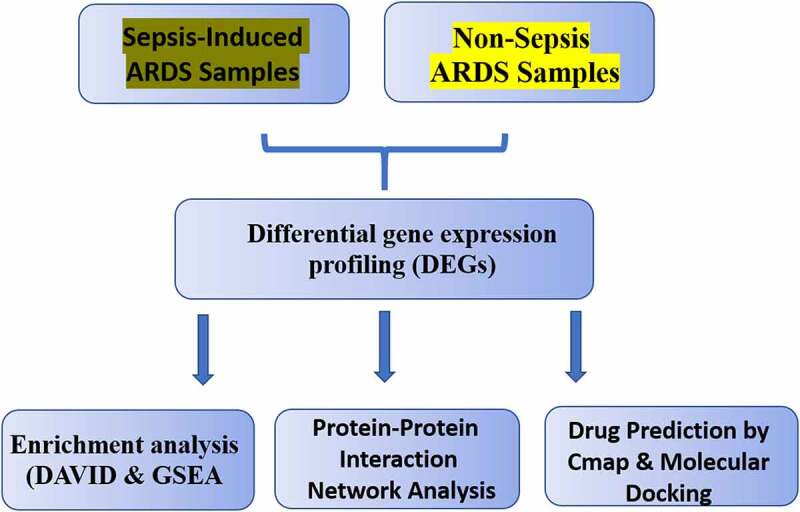

## Introduction

Acute respiratory distress syndrome (ARDS) is a prevalent disease with high mortality, which seriously compromises the quality of life of the patient. Even if the condition of some patients can be ameliorated, there is significant number of sequelae in ARDS survivors, including persistent functional and neurocognitive defects such as cognitive deficits and post-traumatic stress disorder [1]. ARDS imposes health threats to human life quality and is drawing increasing attention. As a major cause of acute respiratory failure, ARDS is a serious consequences of acute inflammatory lung injury. ARDS patients vary in symptom severity and survival longevity, and the identification of mechanisms regulating the variability in disease progression may contribute to the development of efficient personalized treatment [2]. Numerous pathological conditions such as sepsis, pneumonia, multiple blood transfusions, lung contusion, aspiration of stomach contents and drug abuse could result in ARDS. The major predisposing factor is pneumonia or sepsis, which leads to massive neutrophil accumulation within pulmonary vasculatures [3]. Clinical research reveals that sepsis-induced ARDS has poorer recovery from lung injury, higher disease severity and mortality than those induced by other risk factors. Although the research into the pathophysiology of ARDS progresses in recent years, the molecular mechanisms of sepsis-induced ARDS remain to be fully elucidated [4]. On the other hand, in spite of several decades of efforts, there is still a lack of effective pharmacologic interventions to ARDS [5]. The most commonly used medications for ARDS are neuromuscular blocking agents [6], which only functions as an adjuvant to prevent ventilation-related lung injury. Thus, there is an imperative need to develop specific therapeutic drug for treating ARDS.

Over the past decade, research has been focused on the identification of genetic factors correlated with ARDS. With the advancement of molecular biology technology, transcriptome analysis by microarray and RNA-seq become a leading tool for analyzing the global gene expression profile. There are accumulating transcriptome data available in public database such as gene expression omnibus (GEO) [7], and these studies provide novel insights into the pathogenesis of ARDS. For example, study by Kangelaris *et al*. [8] revealed the contribution of neutrophils in progression to ARDS through comparing the transcriptomes between patient with sepsis-induced ARDS and patients with sepsis alone. Another transcriptomic study demonstrated that the elevated expression of interferon-stimulated genes (ISGs) is associated with worse clinical outcomes in ARDS [2]. The recent single cell RNA-seq analysis unveiled distinguishing gene expression profiles in monocyte from patients with sepsis-ARDS [9]. Thorough data mining in transcriptome profile of clinical samples from sepsis-induced ARDS holds great potential to identify bioprocesses and pathways contributing to pathological progression of ARDS, and provide insights into the development of novel therapeutic strategies. However, there is still a lack of systematic data mining workflow for identifying the druggable targets for potential treatment of sepsis-induced ARDS.

In order to survey the molecular mechanism and druggable targets underlying sepsis-induced ARDS, we leveraged the publicly available data and a handful of publicly available database and bioinformatic tools to predict the druggable targets using microarray gene expression data from sepsis-induced ARDS and non-sepsis ARDS samples. Our analysis revealed that anti-microbial infection programs are the key biological process altered in sepsis-induced ARDS. Through protein-protein-interaction (PPI) analysis of the dysregulated genes, we found that TOP2A gene is located at the central hub of the dysregulated gene network, indicating a key regulatory role in sepsis-induced ARDS. Using Connectivity Map (Cmap) database and molecular docking analysis, we further showed that doxorubicin could target TOP2A with a high affinity similar to its endogenous ligand. Collectively, our study benchmarks an *in-silico* strategy to predict the druggable targets and provides novel insights into drug usage in sepsis-induced ARDS. Our *in-silico* analysis framework can be beneficial for other researchers in search for potential treatment targets of other disease conditions.

## Materials and methods

### GEO data acquisition

Gene chip data GSE32707 provided by Dolinay T *et al*. was downloaded firstly from GEO database (*http:www.ncbi.nlm.nih.gov/geo*) in NCBI [10]. GSE32707 data came from the GPL10558 platform and contained 144 patients in the Brigham and Women’s Hospital medical ICU, among which were systemic inflammatory response syndrome (SIRS), sepsis, non-sepsis ARDS (control/untreated), and sepsis-induced ARDS. Given the focus of this analysis was sepsis-induced ARDS, 18 samples from sepsis-induced ARDS patients and 34 untreated samples (non-sepsis ARDS) were retained for the entire analysis. In all the samples, total mRNA was isolated from whole blood samples f on the day of patient admission for microarray analysis. The microarray chip contained 47,220 probes, and 43,951 probes corresponding to 31,326 protein-coding genes were retained. Probes with no detected signals were filtered and the gene level was determined by calculating the median values of multiple probes mapped to the same gene. Finally, expression profiles of 19,565 coding genes in 18 sepsis-induced ARDS samples and 34 untreated samples were retained for the following analysis.

### Analysis of differential-expressed genes (DEGs) between sepsis-induced ARDS and control samples

The DEGs between sepsis-induced ARDS patients and control samples were analyzed using ‘edgeR’ package in R software [11]. The false discovery rate (FDR) and fold change (FC) was calculated. Genes with an FDR< 0.05 and the |log_2_FC| ≥ 1.5 were considered as DEGs.

### Functional enrichment analysis by DAVID

The Database for annotation, visualization and integrated discovery (DAVID, *http://David.abcc.ncifcrf.gov/*) collects and integrates a variety of gene identifiers and more than 40 known publicly available resources, which serves as a comprehensive annotation tool for data mining in transcriptomic data [12]. DEGs between sepsis-induced ARDS group and controls were submitted to DAVID, Gene Ontology (GO) and Kyoto encyclopedia of genes and genomes (KEGG) functional enrichment analysis were performed. Fisher ‘s accurate probability method was selected for of *p* value calculation. Terms with *p* value <0.05 were considered as significantly overrepresented in the DEGs. The ‘ggplot2ʹ package in R software was used for visualizing the Top25 pathways significant enriched in the DEGs.

### Gene set enrichment analysis (GSEA)

GSEA (*http://www.broadinstitute.org/gsea/index.jsp*) computationally determines whether a priori defined gene set shows concordant differences between two biological states [13]. In this study, GSEA was performed with expression profiles of all genes identified in sepsis-induced ARDS and control group. All gene sets in molecular signatures database (MsigDB), including canonical pathways, KEGG gene sets, GO gene sets and GO biological process, were selected for GSEA analysis. According to the default weighted enrichment statistical method, 1000 times’ permutation were selected for each analysis and pathways or GO terms with *p* value <0.05 was considered as significant enrichment gene sets.

### Analysis of protein–protein interaction of DEGs

STRING 9.1 (*http://www.string-db.org/*) database [14] was utilized for identifying the protein–protein interaction network of DEGs with the comprehensive confidence value ≥0.4, which was the default median confidence value in STRING database. The PPI network was visualized using Cytoscape software (http://www.cytoscape.org/).

### Prediction of potential therapeutic drugs for sepsis-induced ARDS

The connectivity map database (Cmap, www.broadinstitute.org/cmap/) was a joint development database of the Massachusetts Institute of Technology (MIT), Harvard University and its affiliated hospitals [15]. At present, Cmap (build 02) contains 6,100 gene expression profiles from 7,056 microarray datasets which cover a total number of 1,309 FDA-approved small molecule drugs. It can be used to predict the mechanisms of actions of novel drugs and perform in-silico screening of existing drugs for drug repurposing. To conduct an in-silico screen of potential therapeutic drugs of sepsis-induced ARDS by querying Cmap, the names of up-regulated and down-regulated genes were converted into standard probes of ‘Affymetrix GeneChip Human Genome U133A Array’ and the query signature format files were constructed according to instructions. The connectivity score has a value between +1 and −1, representing the relative strength of a drug based on the query. Positive connectivity score indicates that a specific drug can induce the expression pattern of sepsis-induced ARDS, and a negative connectivity score indicates the reversion of sepsis-induced ARDS expression pattern. Since we aimed at drug candidates with potential to ameliorate the signatures of sepsis-induced ARDS, the top 20 negatively-regulated drugs were the selected in the downstream analysis.

### Molecular docking

Molecular docking is becoming an important method for drug discovery and structure optimization. Chemical structures of the top 20 candidate drugs were retrieved from the PubChem database and the connectivity map database. Structural format files of SDF were downloaded for subsequent molecular docking. Uniprot was utilized for protein structure query. SystemsDock, a web server for network pharmacology-based prediction and analysis, was used for molecular docking in this study [16]. The PKd/PKi (dissociation constant) was calculated based on in silico molecular docking.

## Results

### Identification of signature genes in sepsis-induced ARDS samples

**Figure 1.**
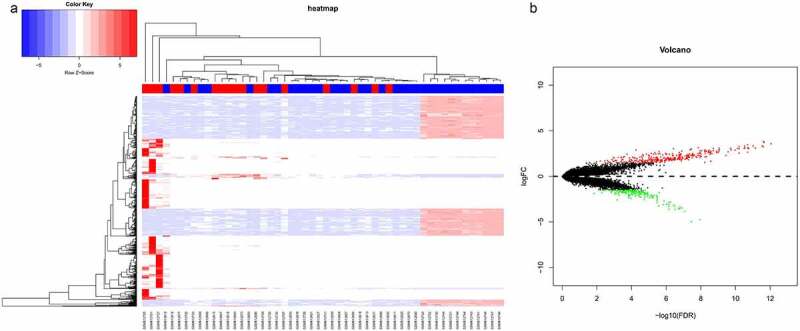
Differential gene expression analysis of sepsis-induced ARDS and control samples. (a) Bidirectional hierarchical clustering of sepsis-induced ARDS and control samples. Each row represented a gene, each column represented a sample. Sepsis-induced ARDS samples are labeled as red and the control samples are labeled as blue. The heatmap shows the Z-score of relative gene expression of each samples; (b) Volcano plot for DEGs between sepsis-induced ARDS and control samples. X-axes indicates -log (FDR) and y-axes showes the log2 fold change. Red dots represent significantly up-regulated genes and green ones represents the down-regulated genes

In order to find genes dysregulated in sepsis-induced ARDS, we retrieved microarray data from GEO database, which contains 18 sepsis-induced ARDS samples and 34 control samples (untreated, non-sepsis ARDS). With a threshold of FDR < 0.05 and |log_2_FC| ≥ 1.5, 330 up-regulated genes and 190 down-regulated genes were identified in sepsis-induced ARDS patients in comparison with control samples. Top 5 up-regulated genes are PURG, ADAMTS6, WDR66, B3GNT7, KRTAP20-3 and the mostly down-regulated genes are CPS1, TM4SF1, GAGE2B, KRT17, NTS (Table 1). All these genes showed a |log2FC| >3, which is equivalent to 8-fold changes in the expression. Bidirectional hierarchical clustering analysis demonstrated that sepsis-induced ARDS samples tend to cluster together, with distinctive gene expression pattern (Figure 1a). Volcano plot demonstrated there are more genes showing significant up-regulation than the down-regulated ones (Figure 1b). We next selected these genes with significant changes (DEGs) as the signature genes for functional enrichment analysis.

**Table 1.** Top ranking list of up-related and down-related genes sepsis-induced ARDS

Gene	logFC	*p*Value	FDR
PURG	3.582800226	4.51E-17	8.82E-13
ADAMTS6	3.740993555	2.45E-16	2.24E-12
WDR66	3.716725258	3.43E-16	2.24E-12
B3GNT7	3.820669571	4.92E-16	2.41E-12
KRTAP20-3	3.236491687	6.48E-16	2.53E-12
CPS1	−4.742218398	3.32E-11	1.10E-08
TM4SF1	−4.994941596	1.60E-10	3.68E-08
GAGE2B	−3.854115188	4.68E-10	8.72E-08
KRT17	−3.462657465	6.84E-10	1.23E-07
NTS	−3.585856923	1.03E-09	1.62E-07

### Functional enrichment analysis revealed altered innate immune responses in sepsis-induced ARDS

**Figure 2.**
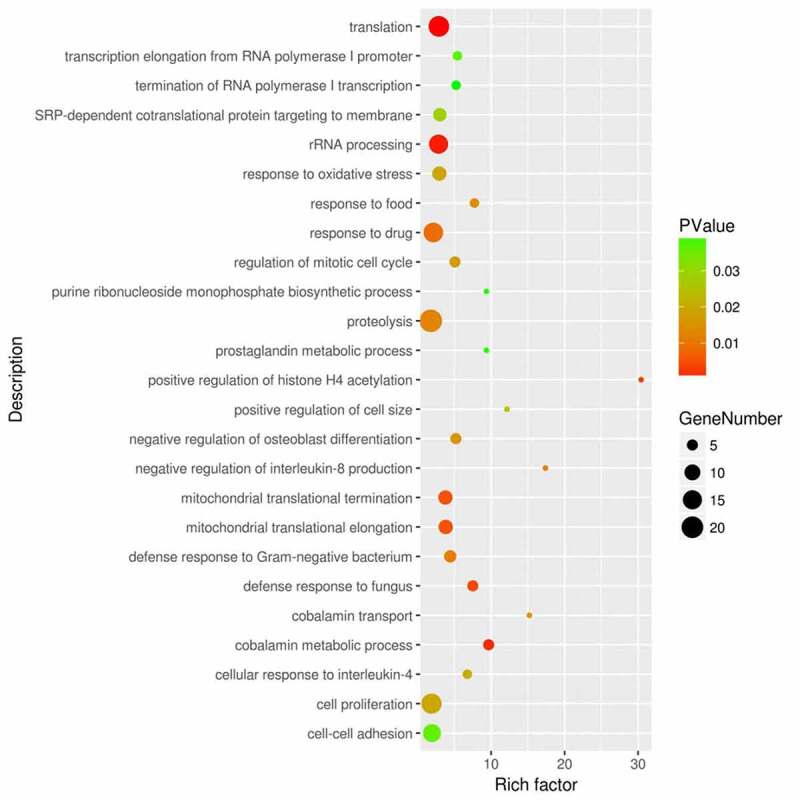
Functional enrichment analysis of the DEGs between sepsis-induced ARDS and control samples. DAVID bioinformatic tools were used for functional enrichment analysis of the DEGs between sepsis-induced ARDS and control samples. Gene number, enrichment score (rich factor) and the enrichment *p* value of each biological process were displayed in the bubble plot

We next performed functional enrichment analysis based on GO and KEGG annotations in DAVID online tools. We identified 32 biological processes that are significantly enriched in the DEGs. Sepsis-induced ARDS seems to affect gene expressions in a wide spectrum of biological processes including translation (GO:0006412), rRNA processing (GO:0006364), cobalamin metabolic process (GO:0009235). Notably, genes involved in innate immunity against pathogens such as defense response to fungus (GO:0050832), defense response to gram-negative bacterium (GO:0050829), cellular response to interleukin-4 (GO:0071353) are also overrepresented in the DEGs (Figure 2). Meanwhile, genes related to the response to oxidative stress (GO:0006979) are also significantly enriched.

**Figure 3.**
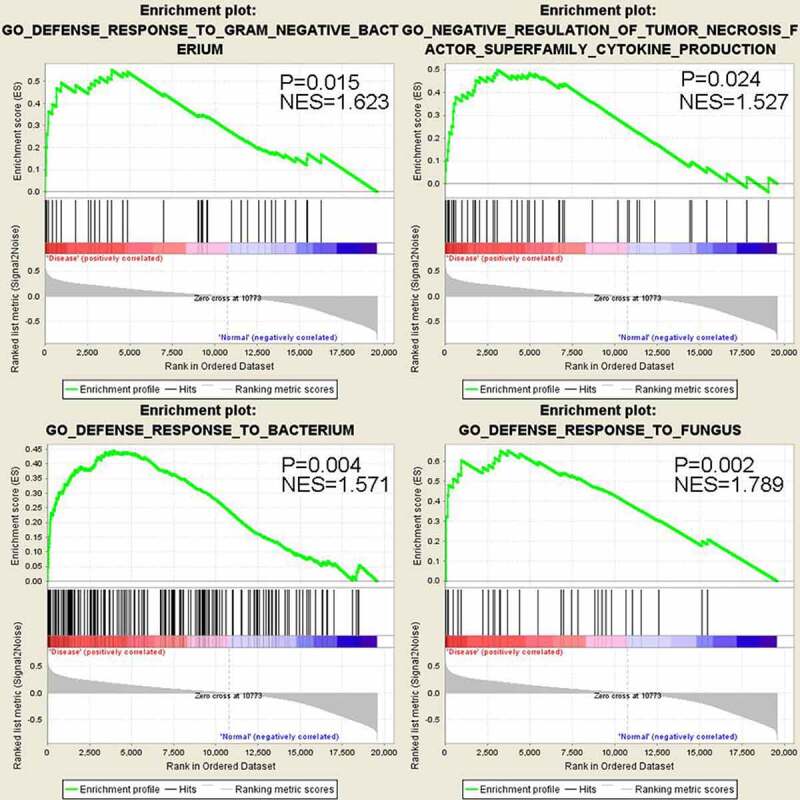
GSEA analysis shows significant up-regulation of genes in anti-bacterial and anti-fungus defense response in sepsis-induced ARDS samples

The DAVID functional enrichment analysis is based on the hypergeometric testing of the overlapping of detected genes and the genes annotated in a specific term, without considering the gene expression level [12]. To incorporate the relative expression information into the enrichment analysis, we next performed Gene Set Enrichment Analysis (GSEA). 23 biological processes were significantly enriched, which also contains innate immunity terms such as ‘defense response to fungus’, ‘defense response to gram negative bacterium’, ‘defense response to bacterium’ and ‘negative regulation of tumor necrosis factor superfamily cytokine production’ (Figure 3). It is important to note that those gene sets show up-regulation (positive correlation) with the sepsis-induced ARDS status. Therefore, GSEA analysis results are highly consistent with that of GO enrichment analysis by DAVID tool, indicating that genes involved in innate immune response against pathogen tend to be up-regulated in sepsis-induced ARDS samples.

### Protein–protein interaction (PPI) analysis identified TOP2A as the top regulator in gene network

**Figure 4.**
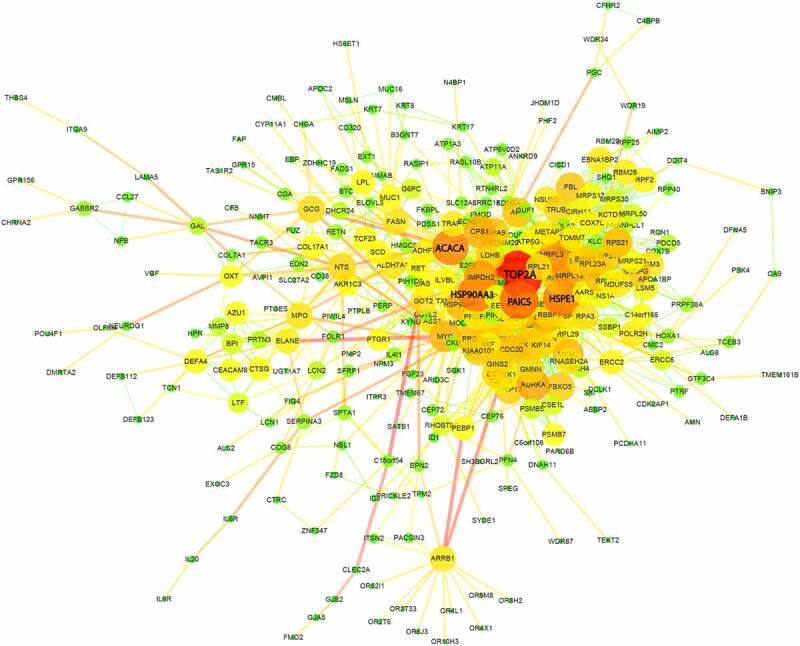
Protein-protein interaction (PPI) network analysis of DEGs between sepsis-induced ARDS and control samples. Each node represents a gene each edge represents an interaction pair. The size and color of each node shows the importance of the gene in the network

To decipher the systematic correlation among the DEGs and the key regulators, we next constructed the PPI network for differentially expressed genes. Based on a combined score >0.4, a total of 332 protein (nodes) with 1,361 protein interaction pairs (edges) were obtained. The PPI network was visualized in the Cytoscape software (Figure 4), which displayed the topological structures for all interactions. Proteins sharing many interaction pairs are located at the hubs, which possibly a critical role in PPI network. The significance degree of the node defined as the number of connections it shares with other proteins is displayed by the size of node. The analysis revealed that five genes (TOP2A, PAICS, HSPE1, HSP90AA1 and ACACA) were at the hubs to interact with many other proteins (Figure 4). Among them TOP2A showed the highest degree of interactions, suggesting that TOP2A gene could be a key regulator in the DEGs network of sepsis-induced ARDS samples.

### In silico *screening of potential therapeutic drugs for sepsis-induced ARDS*

To predict the potential therapeutics from existing drugs for sepsis-induced ARDS treatment, we carried out *in silico* analysis of the DEGs using the Connectivity Map (Cmap) analysis [15]. The rationale is to evaluate the gene expression profile of the existing drugs for its potential to reverse the gene expression patterns in sepsis-induced ARDS samples. Both up- and down-regulated DEGs were submitted in Cmap database as query. Top 20 hits with negative connectivity score (indicating the reversion of sepsis-induced ARDS gene expression pattern) are listed in Table 2. Interestingly, drugs such as doxorubicin, meteneprost, chlorpropamide and trichostatin A were reported to be implicated in controlling fungi or bacterial infections [17–19], suggesting a consistence between the functional enrichment analysis and the drug effect prediction.

**Table 2.** Prediction of potential therapeutic drugs for sepsis-induced ARDS by connectivity map

No.	Cmap name	Dose	Cell	Score	Up	Down
1	SC-19,220	10 µM	PC3	−1	−0.148	0.296
2	meteneprost	10 µM	PC3	−0.982	−0.15	0.286
3	doxorubicin	7 µM	MCF7	−0.913	−0.126	0.279
4	BCB000040	10 µM	PC3	−0.906	−0.136	0.266
5	articaine	12 µM	PC3	−0.892	−0.124	0.272
6	isoflupredone	10 µM	PC3	−0.888	−0.14	0.254
7	vinblastine	100 nM	PC3	−0.879	−0.123	0.267
8	heptaminol	22 µM	PC3	−0.87	−0.149	0.238
9	AR-A014418	10 µM	PC3	−0.864	−0.145	0.239
10	3-acetamidocoumarin	20 µM	PC3	−0.864	−0.172	0.211
11	5,253,409	17 µM	MCF7	−0.86	−0.133	0.249
12	alsterpaullone	10 µM	PC3	−0.859	−0.106	0.275
13	bacampicillin	8 µM	MCF7	−0.858	−0.163	0.218
14	chlorpropamide	100 µM	MCF7	−0.857	−0.108	0.272
15	metampicillin	10 µM	PC3	−0.855	−0.128	0.251
16	estropipate	9 µM	MCF7	−0.852	−0.149	0.229
17	etilefrine	18 µM	PC3	−0.848	−0.138	0.239
18	homatropine	11 µM	MCF7	−0.845	−0.132	0.243
19	trichostatin A	100 nM	MCF7	−0.844	−0.132	0.243
20	fursultiamine	9 µM	MCF7	−0.843	−0.127	0.247

### Molecular docking validated doxorubicin as a high affinity drug for TOP2A

**Figure 5.**
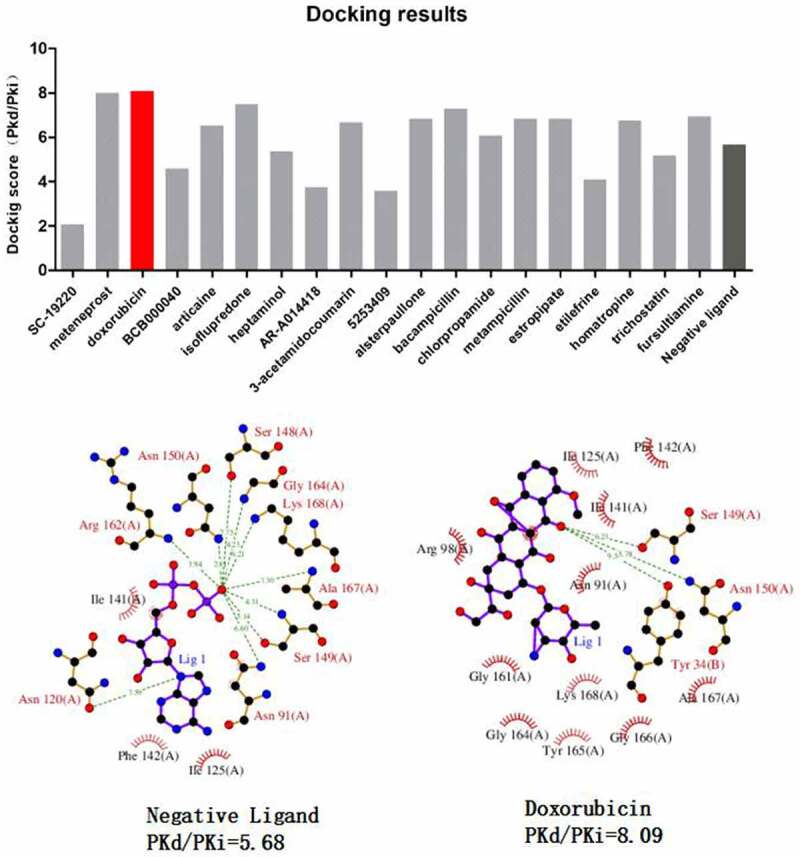
Molecular docking analysis of candidate drugs predicted by Cmap analysis. The lower panel shows the 2D schema chart of TOP2A endogenous ligands and Doxorubicin docking with TOP2A

Our PPI network analysis identified TOP2A as a key regulator in gene network of sepsis-induced ARDS. We hypothesized that some of the top 20 candidate drugs from Cmap analysis may target reverse the gene expression by targeting TOP2A. Therefore, we applied molecular docking analysis to model the interactions between 20 candidate drugs and TOP2A, respectively (Figure 5). We found that doxorubicin showed the highest docking core (PKd = 8.09) with TOP2A. Doxorubicin also displayed a similar affinity toward TOP2A as its endogenous negative ligand. Collectively, our data unveil the potential of doxorubicin as a candidate treatment for sepsis-induced ARDS by targeting TOP2A.

## Discussion

Since the initial description by Ashbaugh et al. in 1967 [20], sepsis-induced ARDS has been recognized as a major clinical problem worldwide, which is posing a high morbidity and mortality burden [21,22]. Although intensive efforts have been made to understand the molecular mechanism of sepsis-induced ARDS, the key defining factors underlying its pathophysiological onset remain elusive [23–25]. Recent studies using single cell RNA-seq approach have revealed the contribution of different immune cell types and immune responses in sepsis-induced ARDS [9,26]. In this study, we utilized the published microarray data from sepsis-induced ARDS samples to probe for the molecular signatures and potential druggable targets. The differential gene expression analysis and function enrichment analysis show that the biological processes of defense to fungal and bacterial infection are overrepresented in the dysregulated genes of sepsis-induced ARDS. These results seem to be consistent with a recent study that RNA-seq analysis from the whole blood sample of ARDS patients shows an elevated innate immunity response [27].

Our PPI network analysis further unveils several key regulators like TOP2A, PAICS, HSPE1, HSP90AA1 and ACACA in the central hubs of dysregulated gene network in sepsis-induced ARDS. TOP2A encodes a Type II DNA topoisomerase which regulates the topological state of DNA during the transcriptional process [28]. It also functions in the process of chromatin modulation in DNA replication and DNA damage repair. Recent studies have reported that TOP2α, as the encoded protein of TOP2A, is the cause of genomic DNA damage [29,30]. Interestingly, DNA damage and repair process has been implicated in a variety of pulmonary diseases, including acute lung injury [31,32]. Since Type II DNA topoisomerase can induce spontaneous double-strand break in genome [29], our data suggest that TOP2A mediated DNA damage response may be involved in the development of sepsis-induced ARDS, which required further experimental validation.

Transcriptome profiling draws increasing attention in drug discovery because it can greatly shorten the time of predicting candidate drugs and mode of actions [33,34]. Lamb *et al*. pioneered the usage of transcriptome data by creating a repertoire of reference transcriptomes of existing drugs [35]. This platform (Cmap) can help reveal the relationship among diseases, drugs and genes, which is of great value in drug repurposing [35,36]. Our analysis using Cmap database identified a variety of candidate drugs for the treatment of sepsis-induced ARDS, including doxorubicin, metampicillin and trichostatin A. Many of these drugs have previously been shown to have effect in controlling fungi or bacterial infections. Since our GSEA analysis results demonstrated that the anti-bacterial and anti-fungal response process are dysregulated in sepsis-induced ARDS, these drugs can potentially reverse the dysregulation of genes involved in anti-fungi or bacterial infection response, thereby ameliorating the conditions of sepsis-induced ARDS. Intestinally, the top-ranked candidate drug doxorubicin is an inhibitor of DNA Topoisomerase II [37] and it also shows high affinity to the top regulator TOP2A protein. Therefore, doxorubicin can be prioritized to evaluate its treatment potential in sepsis-induced ARDS.

Our study is the first to use RNA-Seq data to predict the potential druggable targets in sepsis-induced ARDS. This proof-of-concept study provides insights that are valuable to design future studies to investigate the candidate drugs for treating sepsis-induced ARDS. We also believe that our analysis pipeline using a variety of publicly available database and tools forms a reliable framework for predicting druggable targets based on transcriptome data. However, one confounding factor in the clinical dataset is the heterogeneity amongst different ARDS patients. For example, within the sepsis-induced ARDS samples, we did observe that some samples do not display the same signature gene patterns as the majority. This may be due to the differential immunological status of the patients recruited, and increasing the sample size can reduce the variability introduced by sample heterogeneity. In addition, the expression profiles were extracted from blood cells, which will not capture the changes of lung epithelial cells and endothelial cells. Finally, proteomic analysis of sepsis-induced ARDS samples could generate more insight into the changes at protein level, which can provide solid support of the changes observed at gene expression profiles.

## Conclusions

This study aims to predict the druggable targets of sepsis-induced ARDS based on the analysis of the microarray data. We revealed signatures genes that are significantly dysregulated in sepsis-induced ARDS which may play critical roles in the pathogenesis of sepsis-induced ARDS. Importantly, we further predicted multiple candidate drugs with the potential to reverse the signature of gene expression changes in sepsis-induced ARDS, among which doxorubicin may contribute to reverse the expression mode in ARDS by targeting TOP2A. However, further investigation using ARDS animal model is required to evaluate the *in vivo* effect of these drugs for treat sepsis-induced ARDS treatment. Overall, our analysis benchmarks a workflow for druggable target prediction, which can be widely applicable in other pathological conditions.

## Data Availability

The datasets generated and/or analyzed during the current study are available from the corresponding author on reasonable request.

## Highlights

(1) Transcriptome profiling identified signature genes dysregulated in sepsis-induced ARDS.

(2) GSEA revealed that genes in anti-microbial response is dysregulated in sepsis-induced ARDS.

(3) TOP2A was at the center of dysregulated gene network in sepsis-induced ARDS.

(4) Doxorubicin as a strong candidate targeting TOP2A for reversing transcriptome signature.

## Abbreviation

ARDS: acute respiratory distress syndrome;
PPI: protein-protein interaction
Cmap: Connectivity map
SIRS: Systemic Inflammatory Response Syndrome
DAVID: the Database for annotation, visualization and integrated discovery
GESA: Gene Set Enrichment Analysis
TOP2A: DNA topoisomerase 2-alpha;

## Declaration

**Ethics approval and consent to participate**

Not applicable
